# Epidemiology of tuberculosis in WHO European Region and public health response

**DOI:** 10.1007/s00586-012-2339-3

**Published:** 2012-05-08

**Authors:** Masoud Dara, Andrei Dadu, Kristin Kremer, Richard Zaleskis, Hans H. P. Kluge

**Affiliations:** 1 Communicable Diseases, Health Security and Environment, Tuberculosis and M/XDR-TB Programme, World Health Organization, Regional Office for Europe, Scherfigsvej 8, 2100 Copenhagen, Denmark; 2 Health Systems and Public Health, World Health Organization, Regional Office for Europe, Scherfigsvej 8, 2100 Copenhagen, Denmark

**Keywords:** Pott’s disease, Extra-pulmonary tuberculosis, Multi-drug resistant tuberculosis, European Region, Action Plan

## Abstract

**Purpose:**

To provide an overview of the tuberculosis (TB) and multi-drug resistant tuberculosis (MDR-TB) in the WHO European Region and evolution of public health response with focus on extra-pulmonary tuberculosis and Pott’s disease.

**Methods:**

Authors reviewed regional strategic documents related to TB. The epidemiologic data were reviewed and analyzed.

**Results:**

In the absence of associated pulmonary TB, Pott’s disease is reported as extra-pulmonary TB (up to 47 % of all TB cases in some settings). Due to limitations of the surveillance system, the epidemiology of Pott’s disease and its treatment success are unknown. The Stop TB Strategy and Consolidated Action Plan to Prevent and Combat M/XDR-TB provide comprehensive roadmaps to address all types of TB.

**Conclusions:**

There is a need to further analyze country data to document the extent of Pott’s disease and develop specific guidelines for timely diagnosis and treatment of Pott’s disease.

## Tuberculosis in WHO European Region

**Fig. 1:**
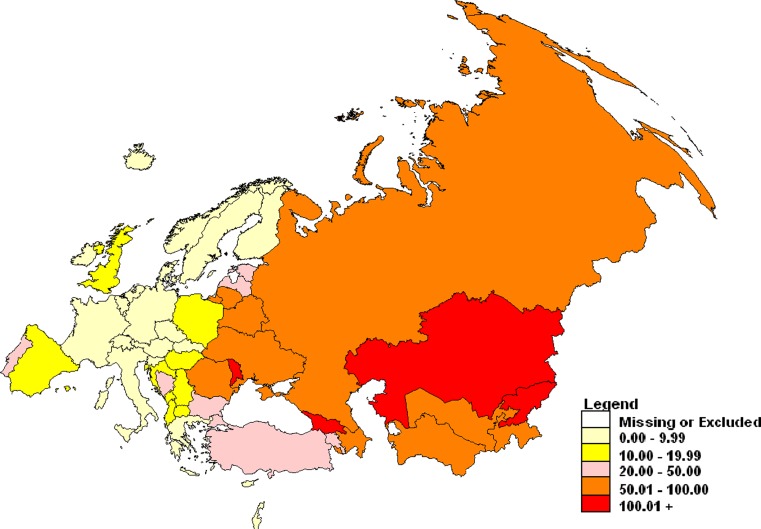
Notification rate of new and previously treated TB patients per 100,000 population, in the WHO European Region in 2010 [1]

The WHO European Region comprises 53 Member States and about 900 million population. The WHO Regional Office for Europe and the European Centre for Diseases Prevention and Control (ECDC) collect tuberculosis (TB) surveillance data on annual basis. There is a wide variation in notification of TB in the Region from 2.8 (Italy) to 123 (Kazakhstan) per 100 000 population (Fig. 1).

**Fig. 2:**
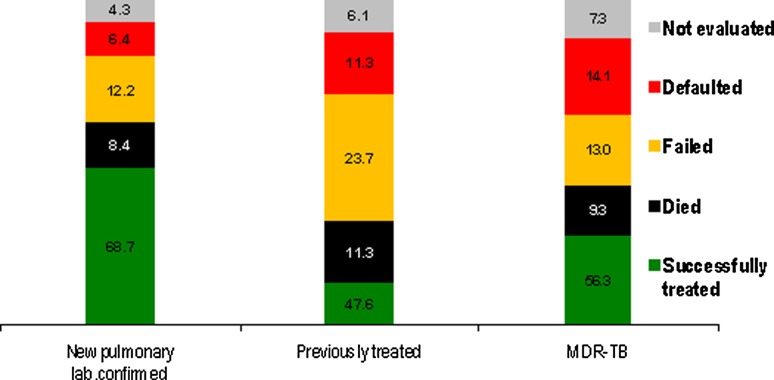
Treatment outcome among new laboratory-confirmed pulmonary TB cases, previously treated cases and MDR-TB cases in the WHO European Region in 2009

MDR-TB is a major issue in the WHO European Region with the percentage of MDR-TB among all TB cases increasing over the last 10 years from 4.3 to 7.5 %. In 2010, 29,000 MDR-TB cases occurred in the Region with 13.7 and 48.7 % MDR-TB among new and previously treated patients, respectively [1]. Extensively drug resistant (XDR) TB amounted 12.2 % in 2010 [1], although this may not be the true magnitude of XDR-TB because of the low coverage of anti-TB drug susceptibility testing for second-line anti-TB drugs, it is certainly expected to be more than 10 %, as was also found in a previous study [14]. The percentage of HIV notified among all TB cases increased from 3.4 to 5.5% in 2008–2010. Over the last 5 years treatment success rates have continued to decrease, falling from 72.5 and 50 % in 2005 to 68.7 and 47.6 % in 2010 among new and previously treated cases, respectively. In 2010, the treatment success rate among MDR-TB patients was 56.3 % (Fig. 2). The high rates of MDR-TB combined with low-treatment outcomes and an increasing HIV epidemic pose an alarming treat to public health in the WHO European Region.

## Extra-pulmonary tuberculosis in the WHO European Region

**Fig. 3:**
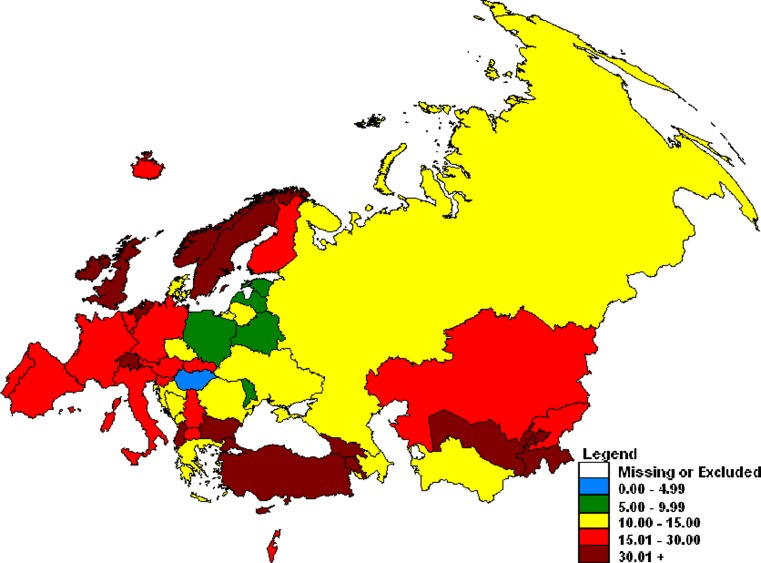
Percentage of extra-pulmonary tuberculosis reported in the WHO European Region in 2010

In 2010, among the 388,875 notified TB cases in the WHO European Region, 65,783 (17 %) cases had extra-pulmonary TB [1]. This proportion of extra-pulmonary cases has remained relatively stable over the last 4 years. Pott’s disease is reported together with other extra-pulmonary TB cases to the joint WHO/ECDC surveillance system and as a result the exact magnitude of the disease is unknown. Country reporting by disaggregated site of disease is good with most countries reporting <1 % of cases with unknown disease localization. Only Turkmenistan, Bosnia and Herzegovina and Denmark still need to improve their notification by disease localization; these countries reported 8.5 %, 11.4 %, and 18.1 % cases with unknown site of disease, respectively. On average, in the European Union/European Economic Area (EU/EEA) countries, a higher proportion of extra-pulmonary localization of the disease among all TB cases was observed than in non-EU/EEA countries; 22 % versus 16 % (Fig. 3). Five countries reported more than 40 % of extra-pulmonary TB cases: the United Kingdom (47 %), the Netherlands (45 %), Andorra (43 %), Norway and Malta (41 % each) [1]. The variances in the reporting of extra-pulmonary TB may result from different diagnostic practices across the region or epidemiological factors, such as immigration or the prevailing *M. tuberculosis* strains [2].

Only for EU/EEA countries anti-TB drug resistance was recorded by site of disease. Among all 31,644 cases with drug susceptibility available in EU/EEA countries, 6,933 were from extra-pulmonary TB cases. The rate of MDR-TB among extra-pulmonary TB cases with drug susceptibility available was 2.3 % (*n* = 158). This rate was lower than the rate of 5.2 % MDR-TB among pulmonary TB cases (*n* = 1,289). The reasons for this need further investigation.

Because of the limited Regional surveillance reporting on data disaggregated by disease localization, further analysis on extra-pulmonary disease, e.g., by HIV status is currently not possible and treatment outcome for cases with this kind of disease is not available.

## Development and implementation of the DOTS Strategy

In 1991, the World Health Assembly recommended that each National Tuberculosis Programme (NTP) work toward two objectives (the “WHO targets”) by the year 2000: (1) to detect at least 70 % of all sputum smear positive cases, and (2) to treat at least 85 % of them successfully, by the introduction of an effective approach to TB control [3] which according to the “World Development Report 1993”, is considered to be one of the most cost-effective health interventions [4].

In 1993, because of resurgence of TB globally, WHO took an unprecedented step and declared TB a global emergency. In 1994, after defining the nature and size of the global TB problem through expanded monitoring and surveillance, the International Union Against Tuberculosis and Lung Disease (The Union) and WHO promoted the technical and managerial approach suggested by Karel Styblo in the 1970s. In 1995, the strategy was packaged and branded as “Directly Observed Treatment Short-course” (DOTS).

The DOTS Strategy incorporating the fundamentals of TB control focused on a five-point policy package [5]:

1. Government commitment to sustained TB control;
2. Sputum smear microscopy to detect the infectious cases;
3. Standardized and directly observed short-course chemotherapy;
4. Uninterrupted supply of medicines;
5. Supervision, Monitoring and Evaluation.

During the period of 1995–2005, the emphasis was on piloting and expanding the DOTS Strategy. DOTS was widely accepted and adopted as a package of essential TB control measures, depending on the peculiarities of certain countries and local circumstances [5, 6].

As a combination of technical and managerial elements, DOTS proved to be effective making the infectious cases as non-infectious and breaking the cycle of transmission. The number of countries using DOTS expanded from only 10 in 1990 to more than 180 in 2005, covering more than 80 % of the world population [7].

After the collapse of the Soviet Union and mainly due to disruption of health services, TB re-emerged in the Region. In response, WHO and other partners scaled up their support for implementation and expansion of DOTS [6]. In 2002, the WHO/Europe Regional Committee endorsed the “DOTS expansion plan to Stop TB in the WHO European Region 2002–2006” [8, 9].

## From DOTS to Stop TB Strategy

The DOTS Strategy has led to major progress in global TB control with successful treatment of nearly 20 million patients worldwide during 1995–2005 [8]. However, new challenges, such as the worsening of the HIV epidemic and rising of TB/HIV co-infection, emergence of drug-resistant strains TB and poor access of most vulnerable population, have proved that DOTS alone will not be sufficient for effective TB control [7, 9–11].

In 2006, WHO developed a new comprehensive strategy for TB control that consists of six components, which builds on and goes beyond DOTS. This strategy addresses the spread of TB and HIV co-infections and MDR-TB [12]. Up to now this is a leading strategy for TB control worldwide, including in the European Region where the rates of M/XDR-TB are the highest in the world and HIV is spreading most rapidly [13–15].

The aim of the Stop TB Strategy is to dramatically reduce the global burden of TB, by halting the epidemic and reversing it by 2015, with the ultimate goal to eliminate TB as a public health problem by 2050. The aims, detailed objectives and targets of the strategy are summarized in Table 1. The six components of the Stop TB Strategy include to (1) pursue high-quality DOTS expansion and enhancement; (2) address TB/HIV, MDR-TB, and the needs of poor and vulnerable populations; (3) contribute to health system strengthening based on primary health care; (4) engage all care providers; (5) empower people with TB, and communities through partnership; (6) enable and promote research. The implementation approaches of these components are listed in Table 2.

**Table 1:** The Stop TB Strategy at a glance

Vision
A TB-free world
Goal
To dramatically reduce the global burden of TB by 2015 in line with the Millennium Development Goals (MDGs) and the Stop TB Partnership targets
Objectives
• Achieve universal access to high-quality care for all people with TB
• Reduce human suffering and socioeconomic burden associated with TB
• Protect vulnerable populations from TB, TB/HIV and drug-resistant TB
• Support development of new tools and enable their timely and effective use
• Protect and promote human rights in TB prevention, care and control
Targets
• MDG 6, Target 6.c: Halt and begin to reverse the incidence of TB by 2015
• By 2015 to reduce prevalence of and deaths due to TB by 50 % compared with a baseline of 1990
• By 2050 to eliminate TB as a public health problem

**Table 2:** Six-components and implementation approaches of the Stop TB Strategy

1. Pursue high-quality DOTS expansion and enhancement • Secure political commitment, with adequate and sustained financing • Ensure early case detection and diagnosis through quality-assured bacteriology • Provide standardized treatment with supervision, and patient support • Ensure effective drug supply and management • Monitor and evaluate performance and impact
2. Address TB/HIV, MDR-TB and the needs of poor and vulnerable populations • Scale-up collaborative TB/HIV activities • Scale-up prevention and management of multidrug-resistant TB (MDR-TB) • Address the needs of TB contacts and of poor and vulnerable populations
3. Contribute to health system strengthening based on primary health care • Help improve health policies, human resource development, financing, supplies, service delivery and information • Strengthen infection control in health services, other congregate settings and households • Upgrade laboratory networks, and implement the practical approach to lung health • Adapt successful approaches from other fields and sectors, and foster action on the social determinants of health
4. Engage all care providers • Involve all public, voluntary, corporate and private providers through public–private mix approaches • Promote use of the International Standards for Tuberculosis Care
5. Empower people with TB, and communities through partnership • Pursue advocacy, communication and social mobilization • Foster community participation in TB care, prevention and health promotion • Promote use of the Patients’ Charter for Tuberculosis Care
6. Enable and promote research • Conduct programme-based operational research • Advocate for and participate in research to develop new diagnostics, drugs and vaccines

DOTS as a five-point package remains the first component and foundation of the Stop TB Strategy. The other components of the Strategy highlight the need to address the challenge of drug-resistant TB and the co-epidemics of TB and HIV, the importance of engaging all care providers in TB care and control and of contributing to strengthening health systems, the role of communities and people with TB, and the fundamental role of research and development for new diagnostics, new drugs and new vaccines.

## Implementation of the Stop TB Strategy and future TB control in the WHO European Region

Evidence-based data suggest that improved diagnosis and treatment based on the Stop TB Strategy have already saved millions of lives globally [16]. The decline of TB incidence is <1 % per year and the long-term elimination target, to reduce incidence to less than one case per million by 2050, will not be reached with existing technologies and approaches [10, 11]. Trend analysis of the MDG targets in 2010 showed that estimated prevalence in the European Region has been declining, but not enough to be able to reach the MDG6 target by the 2015 deadline. Estimated mortality from TB decreased to 6.8 per 100,000 population in 2010. To meet the MDG 6 target, TB mortality must further decline to 6.5 per 100,000 population by 2015 [16].

The WHO Regional Office for Europe held the WHO European Ministerial Forum “All against Tuberculosis” on 22 October 2007 in Berlin, Germany [17], to accelerate progress towards achieving the MDG’s targets for TB control in the WHO European Region. The main outcome of the Forum was the adoption of the Berlin Declaration on TB as well as endorsement of the two Regional plans: the WHO/Europe Plan to Stop TB in 18 High-priority countries in the WHO European Region 2007–2015 and ECDC Framework Action Plan to fight tuberculosis in the European Union [18, 19].

The Forum and the Declaration in particular renewed commitments to take actions to control TB in the European Region highlighting that: (1) TB, and MDR-TB in particular, is a health security threat; (2) Strengthening political and financial commitment is vital to reach the MDGs and (3) to establish adequate fora and mechanisms to assess progress at regional level [18].

It is known that TB is a social disease and it is seen frequently in stigmatized and vulnerable groups such as the poor, migrants, drug abusers, prisoners. Activities are going on currently to reach these groups, addressing social determinants, ethical values and human rights in collaboration with other programmes within WHO as well as national and international partners [10, 11].

## Action Plan to prevent and combat M/XDR-TB in WHO European Region 2011–2015

M/XDR-TB is a man-made phenomenon that emerges as a result of inadequate treatment of tuberculosis and/or poor airborne infection control in health care facilities and congregate settings. The spread of M/XDR TB is also an indication to low adherence to evidence-based TB control practices [13–15, 20, 21].

WHO Regional Office for Europe, in collaboration with technical agencies, Member States, civil society organizations and communities, developed a Consolidated Action Plan to Prevent and Combat M/XDR-TB in WHO European Region 2011–2015. The Consolidated Action plan and its accompanying resolution EUR/RC61/R7 were fully endorsed at the sixty-first session of the WHO Regional Committee for Europe in Baku, Azerbaijan in September 2011 [22]. The plan emphasizes on strengthening the quality of implementation of the Stop TB Strategy, in particular the essential elements of TB control, such as early detection and notification of all TB cases as well as supervised treatment avoiding the misuse of anti-TB drugs [22].

The plan calls for accelerated action, working in close partnership with member states and all partners, such as the Global Fund to Fight AIDS, Tuberculosis and Malaria and the Stop TB Partnership. The goal of the plan is to contain the spread of drug-resistant TB by achieving universal access to prevention, diagnosis and treatment of M/XDR-TB in all member states in the WHO European Region by 2015. The targets of the plan are to diagnose at least 85 % of all estimated MDR-TB patients, treat successfully at least 75 % of all patients notified as having MDR-TB and to decrease by 20 % points the proportion of MDR-TB among previously treated patients [21].

The Consolidated Action plan to prevent and combat M/XDR-TB comprises six strategic directions and seven areas of intervention. The strategic directions are cross-cutting and are designed to safeguard the values of the Health 2020 strategy. These include: (1) identifying and addressing determinants and underlying risk factors contributing to the emergence and spread of drug-resistant TB; (2) strengthening the health system response in providing accessible, affordable and acceptable services using patient-centered approaches; (3) working in national, regional and international partnerships on TB prevention, control and care; (4) fostering regional and international collaboration for development of new diagnostic tools, medicines and vaccines against TB; (5) promoting rational use of existing resources, identifying gaps and mobilizing additional resources to fill the gaps; (6) monitoring the trends of M/XDR-TB in the Region and measuring the impact of interventions.

The areas of intervention are in line with the Global Plan to Stop TB 2011–2015 and include the same targets as set by the Global Plan and World Health Assembly resolution WHA62.15, namely to provide universal access to diagnosis and treatment of MDR-TB: (1) prevent the development of M/XDR-TB cases; (2) scale up access to testing for resistance to first- and second-line anti-TB drugs and to HIV testing and counseling among TB patients; (3) scale up access to effective treatment of drug-resistant TB; (4) scale up TB infection control; (5) strengthen surveillance, including recording and reporting, of drug-resistant TB; (6) expand country capacity to scale up the management of drug-resistant TB, including advocacy, partnership and policy guidance; (7) address the needs of special populations.

The action plan clearly defines milestones and a detailed set of recommended activities. Furthermore, a monitoring framework was developed to track progress toward milestones and the eventual achievement of objectives.

To address the lack of international consensus on the role of surgery in pulmonary and extrapulmonary TB including tuberculosis spondylitis, the plan foresees that the WHO Regional Office in collaboration with the Member States and other partners develop a set of evidence-based criteria for surgery for M/XDR-TB patients by the end of 2012.

**Fig. 4:**
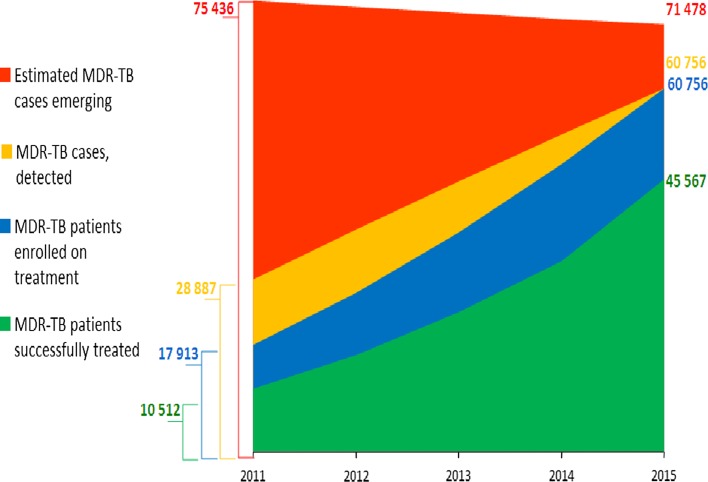
Expected achievements from implementation of the Consolidated Action Plan to present and combat M/XDR-TB in the WHO European Region, 2011–2015 [21]

If the Plan is implemented, 225,000 MDR-TB patients will be detected and 127,000 of them will be successfully treated (Fig. 4). As a result of successful implementation of the Plan, 250,000 MDR-TB and 13,000 XDR-TB cases would be averted and 120,000 lives will be saved. The direct economic gain in lives saved by the Plan amounts to US$ 5 billion over the 5 years. In addition, US$ 7 billion will be saved directly on costs for detection and care of the M/XDR-TB cases averted, which would have arisen and needed treatment in the absence of improved TB control provisions of the plan. Its implementation will also have an impact on preventing transmission, and thus averting many more MDR-TB cases beyond 2015 that are as yet undetermined, but will go far beyond this number.

## Conclusion

TB is still causing a considerable health burden in the WHO European Region, with increasing MDR-TB rates. There is a need to improve surveillance and/or conduct surveys to get a better understanding on the extent of Pott’s disease and the reasons for the wide variation of the proportion of extra-pulmonary TB among all TB cases across the Region. Countries need to urgently scale up the implementation of the Stop TB Strategy, ensuring early diagnosis and proper treatment, strengthening health-system policies, establishment of links with the broad economic and health reforms, including addressing social determinants of TB, and promotion of research efforts, including the development of new diagnostics, anti-TB drugs and vaccines.

Adequate interventions addressing drug-resistant TB require proper national planning and effective implementation of comprehensive approaches with the support from national and international partners. Further research on optimal treatment and care for Pott’s disease is needed.
